
JOURNAL INFORMATION
==============================
NLM Title Abbreviation: Tex Med J (Austin)
NLM Journal ID: 21130590R

Texas Medical Journal

ISSN: 0892-8495
Publisher: Biomedical Journal Digitization Project

ARTICLE INFORMATION
==============================
PMCID: PMC9591664
Article version: 1
Subjects: Books and Magazines

Rhinology, Laryngology and Otology, and Their Significance in General Medicine

Print publication date: 1901 Jan
Volume: 16
Issue: 7
First page: 322
Last page: 322
License: This work is free of known copyright restrictions. For more information, please see PMC Back Issue Digitization.
License URL: https://creativecommons.org/publicdomain/mark/1.0/

==============================

---

RHINOLOGY, LARYNGOLOGY AND OTOLOGY, AND THEIR SIGNIFICANCE IN GENERAL MEDICINE, by E. P. Friedrich, M. D., Privadocent at the University of Leipzig. Authorized translation from the German, Edited by H. Holbrook Curtis, M. D., consulting Surgeon to the New York Nose and Throat Hospital and to the Diphtheria and Scarlet Fever Hospitals. 348 pages—price $2.50. Philadelphia and London, W. B. Saunders & Co., Publishers.

The physician who would familiarize himself with the diseases of the ear, nose and throat, will find this a book of inestimable value. The relation of these diseases to those of the general system have been too generally disregarded, and while every year brings out new publications which serve, in a measure, the purpose of the specialist, but few books have been written that are adapted to the physician who is seeking what is to him practical information along this line of work.

The relations existing between the nose, throat and ear are set forth in detail, as are also the diseases of these organs to those of the general system.

Early symptomatic manifestations in the respiratory tract, reflex neuroses of the ear and air-passages and their diagnostic significance receive a full share of consideration. The chapters on the exanthemata and their sequlæ are exceptionally interesting and rich in new material.

The book deserves the commendation it is receiving at the hands of the profession, and every physician and specialist can well afford to make it a part of his library. W. A. H.

---

**A Few Left.—A copy of Dr. Daniel's book, "Recollections of a Rebel Surgeon," will be given as a premium for new subscribers to the Texas Medical Journal for 1901. Send 10 cents extra for postage on book. Subscription $1.**
